# Electronic Cigarette Use Among Middle and High School Students — United States, 2011–2012

**Published:** 2013-09-06

Electronic cigarettes, or e-cigarettes, are battery-powered devices that provide doses of nicotine and other additives to the user in an aerosol. Depending on the brand, e-cigarette cartridges typically contain nicotine, a component to produce the aerosol (e.g., propylene glycol or glycerol), and flavorings (e.g., fruit, mint, or chocolate) (1). Potentially harmful constituents also have been documented in some e-cigarette cartridges, including irritants, genotoxins, and animal carcinogens (1). E-cigarettes that are not marketed for therapeutic purposes are currently unregulated by the Food and Drug Administration, and in most states there are no restrictions on the sale of e-cigarettes to minors. Use of e-cigarettes has increased among U.S. adult current and former smokers in recent years (2); however, the extent of use among youths is uncertain.

Data from the 2011 and 2012 National Youth Tobacco Survey (NYTS), a school-based, pencil-and-paper questionnaire given to U.S. middle school (grades 6–8) and high school (grades 9–12) students, were used to estimate the prevalence of ever and current (≥1 day in the past 30 days) use of e-cigarettes, ever and current (≥1 day in the past 30 days) use of conventional cigarettes, and use of both. NYTS consists of a cross-sectional, nationally representative sample of students in grades 6–12 from all 50 states and the District of Columbia (3).

During 2011–2012, among all students in grades 6–12, ever e-cigarette use increased from 3.3% to 6.8% (p<0.05) (Figure); current e-cigarette use increased from 1.1% to 2.1% (p<0.05), and current use of both e-cigarettes and conventional cigarettes increased from 0.8% to 1.6% (p<0.05). In 2012, among ever e-cigarette users, 9.3% reported never smoking conventional cigarettes; among current e-cigarette users, 76.3% reported current conventional cigarette smoking.

Among middle school students, ever e-cigarette use increased from 1.4% to 2.7% during 2011–2012 (p<0.05) (Figure); current e-cigarette use increased from 0.6% to 1.1% (p<0.05), and current use of both e-cigarettes and conventional cigarettes increased from 0.3% to 0.7% (p<0.05). In 2012, among middle school ever e-cigarette users, 20.3% reported never smoking conventional cigarettes; among middle school current e-cigarette users, 61.1% reported current conventional cigarette smoking.

Among high school students, ever e-cigarette use increased from 4.7% to 10.0% during 2011–2012 (p<0.05) (Figure); current e-cigarette use increased from 1.5% to 2.8% (p<0.05), and current use of both e-cigarettes and conventional cigarettes increased from 1.2% to 2.2% (p<0.05). In 2012, among high school ever e-cigarette users, 7.2% reported never smoking conventional cigarettes; among high school current e-cigarette users, 80.5% reported current conventional cigarette smoking.

E-cigarette experimentation and recent use doubled among U.S. middle and high school students during 2011–2012, resulting in an estimated 1.78 million students having ever used e-cigarettes as of 2012. Moreover, in 2012, an estimated 160,000 students who reported ever using e-cigarettes had never used conventional cigarettes. This is a serious concern because the overall impact of e-cigarette use on public health remains uncertain. In youths, concerns include the potential negative impact of nicotine on adolescent brain development (4), as well as the risk for nicotine addiction and initiation of the use of conventional cigarettes or other tobacco products.

CDC and the Food and Drug Administration will continue to explore ways to increase surveillance and research on e-cigarettes. Given the rapid increase in use and youths’ susceptibility to social and environmental influences to use tobacco, developing strategies to prevent marketing, sales, and use of e-cigarettes among youths is critical.

## Reported by

*Catherine Corey, MSPH, Baoguang Wang, MD, Sarah E. Johnson, PhD, Benjamin Apelberg, PhD, Corinne Husten, MD, Center for Tobacco Products, Food and Drug Administration. Brian A. King, PhD, Tim A. McAfee, MD, Rebecca Bunnell, PhD, René A. Arrazola, MPH, Shanta R. Dube, PhD, Office on Smoking and Health, National Center for Chronic Disease Prevention and Health Promotion, CDC.* ***Corresponding contributor:*** *Brian A. King, baking@cdc.gov, 770-488-5107.*

## Figures and Tables

**FIGURE:**
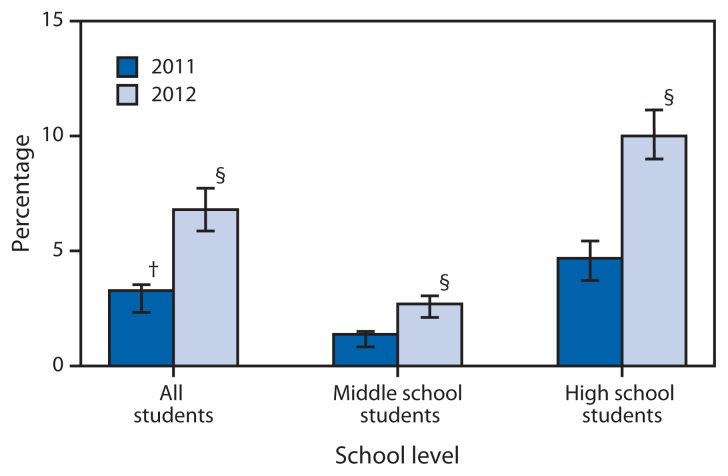
Ever electronic cigarette use^*^ among middle and high school students, by year — National Youth Tobacco Survey, United States, 2011–2012 ^*^ Ever electronic cigarette use defined as having ever used electronic cigarettes, even just one time. ^†^95% confidence interval. ^§^ Statistically significant difference between 2011 and 2012 (chi-square, p<0.05).
